# Integrated transcriptomes throughout swine oestrous cycle reveal dynamic changes in reproductive tissues interacting networks

**DOI:** 10.1038/s41598-018-23655-1

**Published:** 2018-04-03

**Authors:** Jun-Mo Kim, Jong-Eun Park, Inkyu Yoo, Jisoo Han, Namshin Kim, Won-Jun Lim, Eun-Seok Cho, Bonghwan Choi, Sunho Choi, Tae-Hun Kim, Marinus F. W. te Pas, Hakhyun Ka, Kyung-Tai Lee

**Affiliations:** 1 Department of Animal Science and Technology, Chung-Ang University, Anseong, Gyeonggi-do 17546 Republic of Korea; 2 Animal Genomics and Bioinformatics Division, National Institute of Animal Science, Rural Development Administration, Wanju, 55365 Republic of Korea; 3 Division of Biological Science and Technology, Yonsei University, Wonju, 26493 Republic of Korea; 4 Personalized Genomic Medicine Research Center Genome Institute, Korea Research Institute of Bioscience and Biotechnology, Daejeon, Republic of Korea; 5 Animal Breeding and Genomics, Wageningen UR Livestock Research, 6700AH Wageningen, The Netherlands

## Abstract

Female fertility is a highly regulated process involving the synchronized activities of multiple tissues. The underlying genomic regulation of the tissue synchronization is poorly understood. To understand this better we investigated the transcriptomes of the porcine ovary, endometrium, and oviduct at days 0, 3, 6, 9, 12, 15, or 18 of the oestrous cycle. We analysed the transcriptome profiles of the individual tissues and focus on the bridging genes shared by two or more tissues. The three tissue-networks were connected forming a triangular shape. We identified 65 bridging genes with a high level of connectivity to all other genes in the network. The expression levels showed negative correlations between the ovary and the other two tissues, and low correlations between endometrium and oviduct. The main functional annotations involved biosynthesis of steroid hormones, cell-to-cell adhesion, and cell apoptosis, suggesting that regulation of steroid hormone synthesis and tissue viability are major regulatory mechanisms.

## Introduction

Comprehensive understanding of the regulatory mechanism of the oestrous cycle in domestic animals is important for manipulating animal productivity and resolving reproductive problems such as infertility^1^. This is of particular interest in pigs, for which reproductive rate and litter size impact commercial value. The complicated range of hormone secretions and physiological changes that appear during the oestrous cycle are closely linked to multiple molecular interactions among a number of reproduction-related tissues, including the hypothalamus, the pituitary, the ovary, the oviduct, and the endometrium^2^. The oestrous cycle lasts an average of 21 days in female pigs. At the start of the oestrous cycle, levels of estrogen are highest and the release of pre-ovulatory gonadotrophin releasing hormone (GnRH) and luteinising hormone (LH) occurs as a result of the positive feedback mechanism of estrogen action, and ovulation follows. Then, levels of progesterone begin to increase after ovulation and decrease at late diestrus when the implanting conceptuses are not present in the uterus and luteolysis is induced.

The cyclic and sequential secretion of hormones from each reproductive tissue regulates morphological and functional changes in reproductive tissues to mediate the progression of the oestrous cycle^3,4^. The changes in hormonal milieu control ovarian activity which leads to follicular development, ovulation, luteinisation and luteolysis^5^. The ovarian activity governs the oviductal function for the sperm capacitation, fertilization and preimplantation embryo development, and the endometrial function regulatory for conceptus implantation and the establishment and maintenance of pregnancy^6–8^. In addition, the ovary acts on the hypothalamus and the pituitary glands at a positive and negative feedback mechanism for regulation of hormone production of those tissues. The uterine endometrium is involved in regulation of ovarian function by producing the luteolytic signal, prostaglandin F_2α_(PGF_2α_), at the end of the oestrous cycle in domestic animal species. Thus, the cyclicity of the oestrous cycle is the result of complicated but well-coordinated reciprocal interactions among reproductive tissues^9–11^. However, the regulatory mechanism of the oestrous cycle at the cellular and molecular levels and the expression and function of genes in reproductive tissues are not fully understood.

Transcriptome changes in the ovary, the endometrium, and the oviduct have been studied under different experimental conditions. The oviductal transcriptome has shown to be influenced by the ovarian hormones such as estrogen and progesterone^12,13^. Ovarian transcriptome analyses revealed genes that related to litter size in pigs^14^. In addition, transcriptomic changes in the endometrial tissue have been analysed at the time of implantation and during pregnancy^15,16^. In humans, many studies have focused on the changes in the transcriptomes by the presence of the embryo or due to reproductive failure in the endometrium and oviduct^17,18^. However, it has not been attempted to investigate the dynamic regulatory mechanism in the transcriptomes and the coordination of the transcriptome changes in multiple reproductive tissues during different phases of the reproductive cycle in any animal species.

The objective of this study was to investigate the molecular mechanisms underlying the synchronization of the ovary, endometrium and oviduct reproductive tissues in the pig. We identified the differentially expressed genes during the different phase of the oestrous cycle and among different reproductive tissues and the key genes in each network to elucidate the biological roles of these genes in the reproductive tracts. Then, we integrated the transcriptomes of the ovary, the endometrium, and the oviduct and analyse both universal and tissue-specific gene regulation throughout the oestrous cycle using RNA-Seq analysis. Dynamic molecular networks from the integrated transcriptomes were elucidated by the gene co-expression network (GCN) analysis. Here, we report that differentially expressed genes shared among the reproductive tissues in a universal and tissue-specific manner may be involved in regulating the cyclicity of the oestrous cycle in pigs.

## Results

### Integration of reproductive tissue transcriptomes during the oestrous cycle

**Figure 1:**
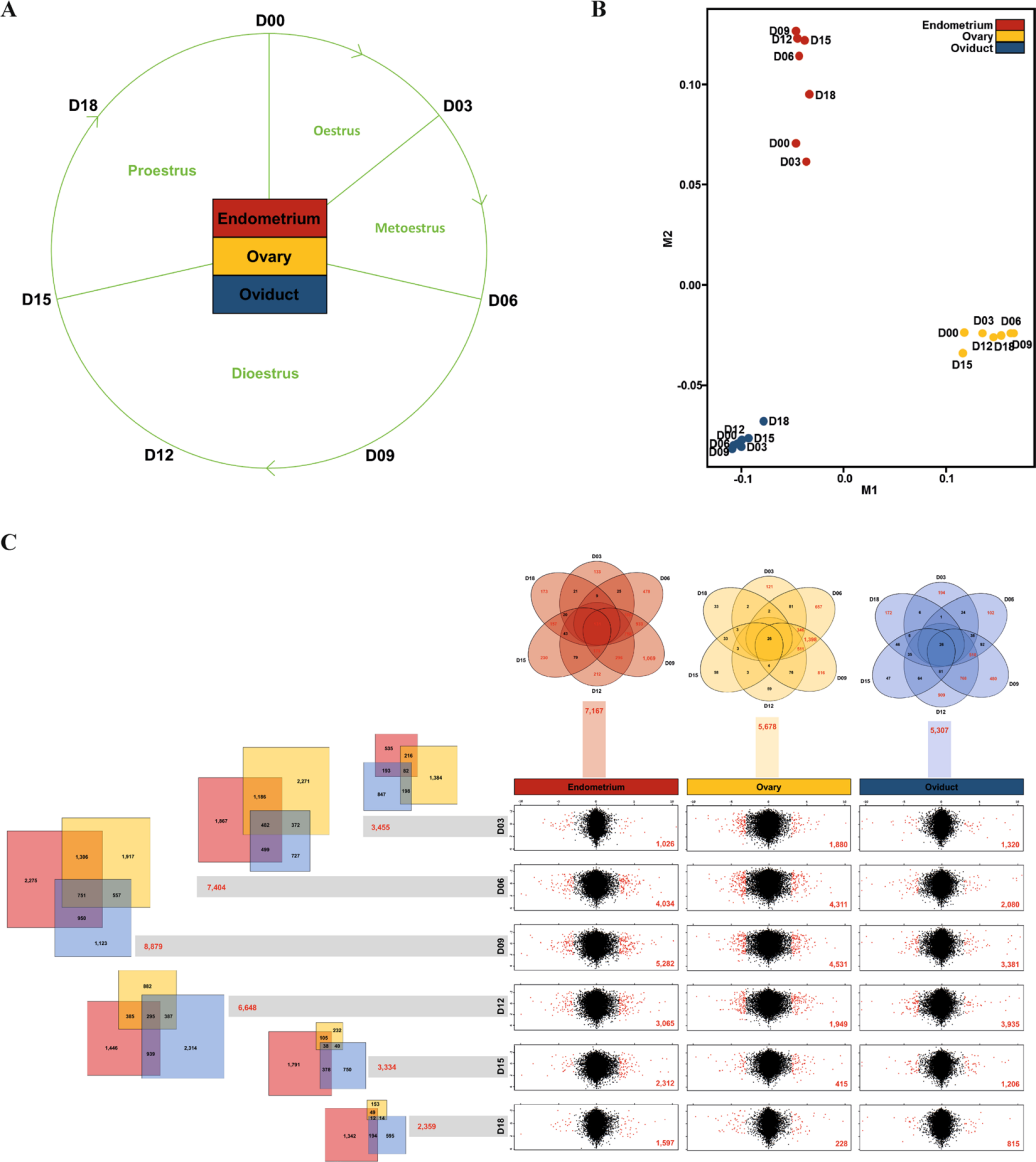
Overview of reproductive tissue transcriptomes by passing through a swine oestrous cycle. (**A**) Transcriptomes in three female reproductive tissues (endometrium, ovary and oviduct) for 7 dates (day 0, D00; day 3, D03; day 6, D06; day 9, D09; day 12, D12; day 15, D15 and day 18, D18) on the phases of the oestrous cycle. (**B**) A principal component analysis revealed far distances between different tissue groups and clusters among the seven time points transcriptomes based on the oestrous cycle within tissue groups. (**C**) Dynamic view of DEGs based on the start of oestrous cycle (D00). The x-y axes of scatter plots were scaled by log10 and log2 fold changes. Significant DEGs (FDR < 0.05) in the scatter plots were coloured and counted by *red* dots and a number. Venn diagrams with scaled bars revealed numbers of integrated DEGs by each of time points and tissues. The numbers over 100 in the tissue specific Venn diagrams were indicated by *red* colour.

We obtained reproductive tissues from the endometrium, ovary, and oviduct, and analysed the transcriptomes expressed in those tissues on days 0, 3, 6, 9, 12, 15, and 18 of the oestrous cycle (Fig. 1A). The RNA-seq data revealed clear differences among the three tissues and the transcriptomes from different time points throughout the oestrous cycle were clustered within individual tissues (Fig. 1B). To better understand the dynamic changes of each tissue-specific transcriptome throughout the oestrous cycle, we compared transcriptomes of each tissue at each time point to the transcriptome of corresponding tissue on day 0 and determined the differently expressed genes (DEGs) (Fig. 1C). Large changes in gene expression were observed between day 6 and day 9 in the endometrial and ovarian transcriptomes and between day 9 and day 12 in the endometrial and oviductal transcriptomes (DEGs > 3,000 transcripts). We also compared the number of significant DEGs among the three tissues at each time point. The Venn diagrams representing the overlap DEGs between the tissues at each time point revealed that the number of overlap DEGS increased until day 9 and then rapidly decreased at the end of the cycle indicating genomic regulation of the reproduction processes.

### GCN of the porcine oestrous cycle

**Figure 2:**
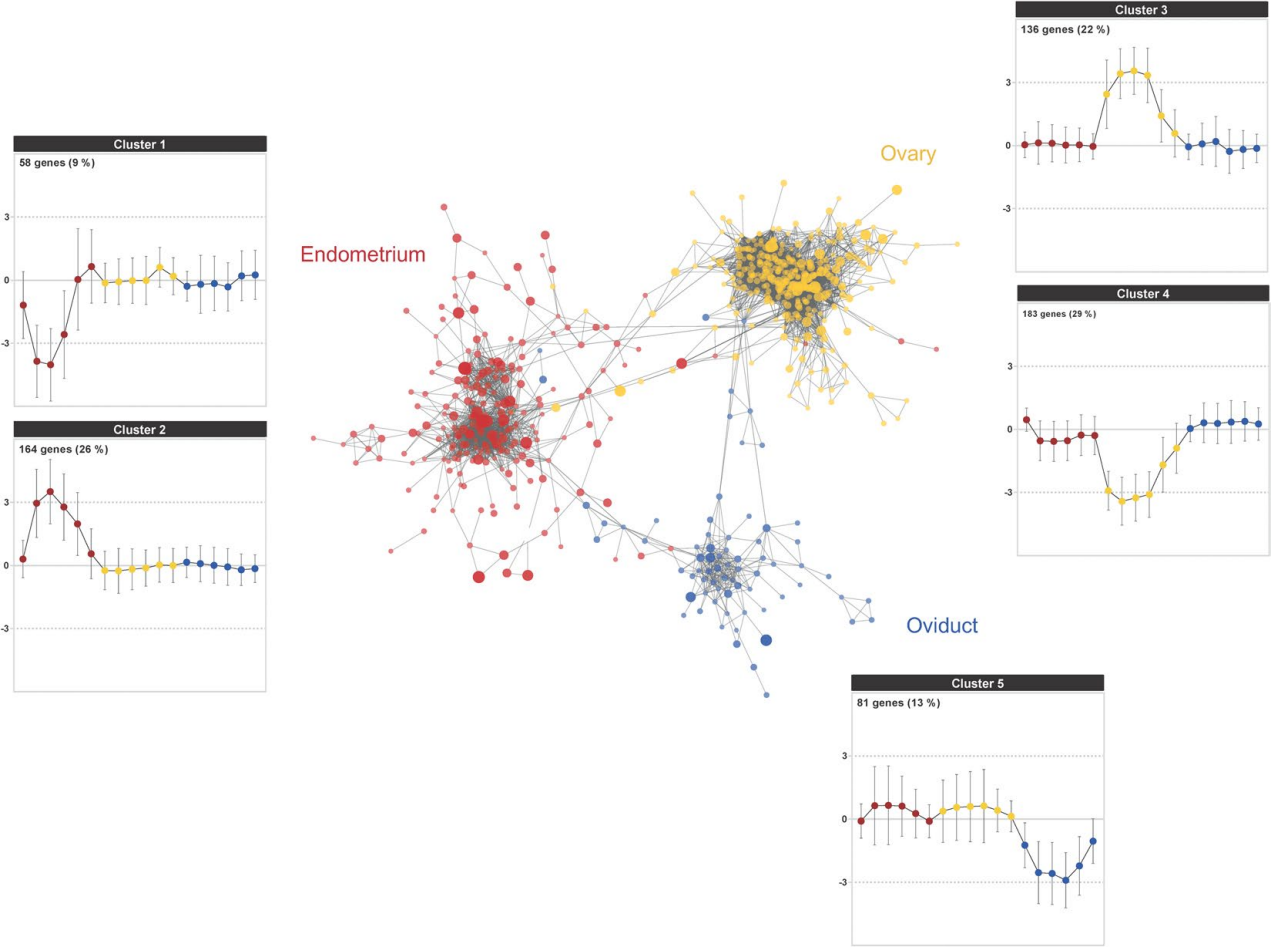
GCN of the porcine oestrous cycle. The network shows three substantial core groups (represented by different node colours), which were clearly separated by each reproductive tissue: endometrium (*red*), ovary (*yellow*), and oviduct (*blue*). The PCIT algorithm generated significant connections and a nominal threshold (*r*^2^ > 0.90) was applied to the network analysis. The overall GCN contained 622 genes connected by 9,639 edges. Node size and transparency were mapped to the highest log2 FC at each time point. Edge transparency was mapped in-between nodes. Gene clustering analysis by the k-means clustering algorithm in the MultiExperiment Viewer software revealed five distinct expression profiles across the oestrous phases (left to right) and tissues (same colour with node). The levels of expression were presented as 3 and −3 log2 FC. The total number of genes and the percentages in the brackets are located at the top left corner in the cluster.

Next, we integrated the entire transcriptomes from three reproductive tissues at seven time points during the oestrous cycle using GCN analysis to study DEG shared by two or three tissues at the same time points. A total of 904 genes were employed in the PCIT algorithm that combines the concept partial correlation coefficient with information theory to estimate significant connections between genes. A GCN was constructed with 622 nodes (genes) and the nodes were connected by 9,639 edges (Fig. 2). The network was comprised of three clearly clustered tissue-specific sub-networks representing each tissue with a small number of genes connecting the sub-networks. The sub-networks also revealed different expression patterns based on k-mean expression clustering analysis (Supplementary data 2). We observed that an endometrium-specific sub-network (*red*) showed changes in gene expression between different time points of the oestrous cycle, while the other tissues did not show any changes in gene expression. Moreover, the two different expression clusters in the endometrium represented a sub-network of upregulated genes and a sub-network of downregulated genes, which appeared between days 6 and 12. In the ovary-specific sub-network (*yellow*), the expression changes were noticeable between days 3 and 12, with both up- and downregulations represented as clusters 3 and 4, respectively. The oviduct-specific sub-network had one expression cluster with downregulated genes, whereas the other two tissues showed a slightly upregulated expression pattern.

**Figure 3:**
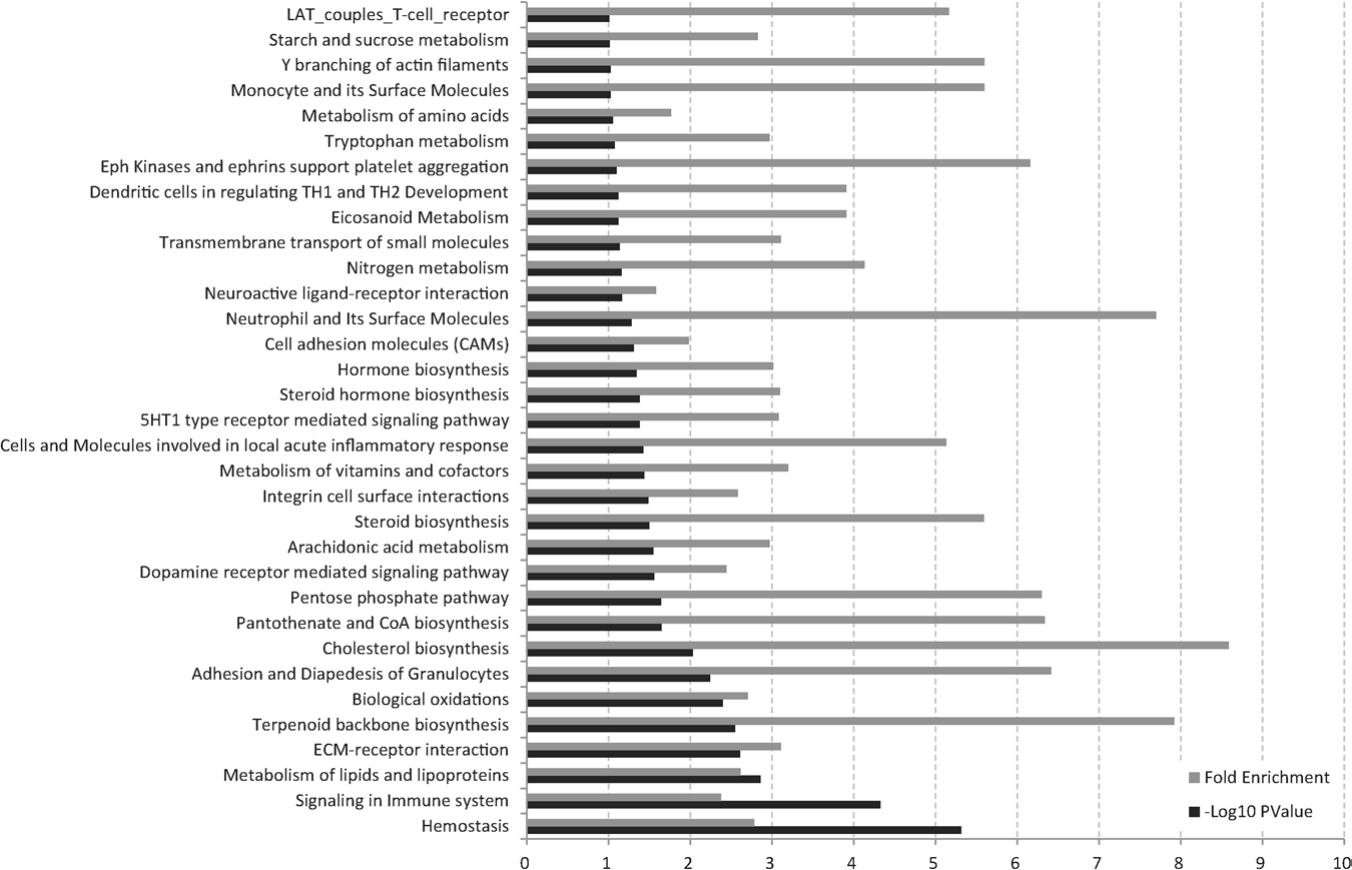
Enrichment analyses of biological meaning in the network. Enrichment analyses of the pathways from all databases in DAVID. The significant pathways in the chart passed a cut-off (−log10 *P-*value > 1.0).

The DAVID enrichment analysis for KEGG pathway terms showed significantly enriched pathways (Fig. 3). There was an enrichment of genes in the network that are a part of several biological pathways involved in the female reproductive system, including haemostasis, signalling in immune system, metabolism of lipids and lipoproteins, extracellular matrix-receptor interaction, and terpenoid backbone biosynthesis^19,20^. In addition, several pathways related to metabolism of prostaglandins and steroid hormones were included. These pathways are important components of the immune protection of the female reproductive tract and affect fertilization and pregnancy success.

### Tissue-specific networks and functional annotation in the oestrous cycle

**Figure 4:**
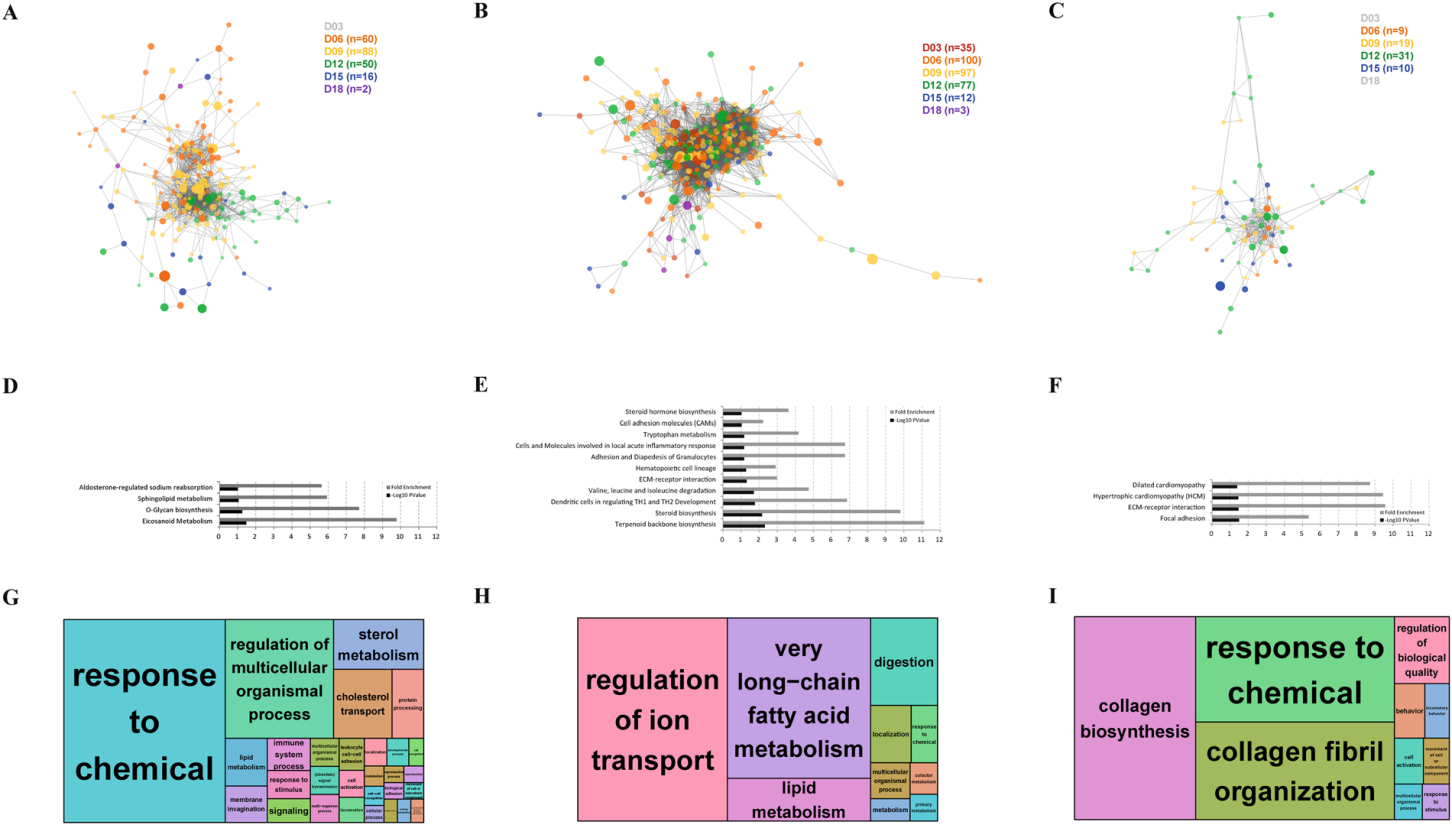
Tissue-specific sub-networks. Sub-networks were reconstructed from the original network in Fig. 2 and enrichment analyses for the genes in the subnetworks were performed. Endometrium (**A**,**D**, and **G**), ovary (**B**,**E**, and **H**), and oviduct (**C**,**F**, and **I**). (**A**–**C**) Differently coloured nodes represent the expression values among the oestrous cycle phases and their values are shown in brackets. The number of nodes for each colour is shown in brackets. (**D**–**F**) DAVID-enriched pathways for the tissue-specific subnetworks after applying the cut-off (−log10 *P*-value > 1.0 and the number of responsible genes > 3). (**G**–**I**) Gene Ontology treemaps regarding biological process terms for the three reproductive tissues. Enriched GO terms are indicated by bold letters.

Based on the presence of tissue-specific sub-networks, we integrated the biological differences and similarities among three reproductive tissues throughout the oestrous cycle. First we investigated the tissue-specific sub-networks separately. Genes were excluded from the independent network if they were not directly connected within the main sub-networks. We provided list of genes involved in each of tissue-specific sub-networks (Supplementary data 3). The independent networks represent the connections between genes that showed the largest FC values in the different phases of the oestrous cycle (Fig. 4A–C).

The endometrium-specific network consisted of 216 DEGs with 1,501 significant connections (r2 > 90). In the endometrium-specific network, day 9 had the highest number of genes with the maximum FC level (D09, n = 88), followed by day 6 (D06, n = 60) and day 12. There were genes that showed the maximum FC level from day 3 until the end of the oestrous cycle and there were only a small number of genes showing the maximum FC level on day 18 (D12, n = 50), as shown in Fig. 4A. The ovary-specific network was the largest of the three tissues, consisting of 324 genes with 7,794 connections (Fig. 4B). In the ovary-specific network, day 6 had the highest number of genes with the maximum FC level (n = 100), followed by day 9. Day 12 showed a high number of nodes, while day 15 and day 18 showed low FC levels. The oviduct-specific network was small compared with the other two networks, consisting of 69 genes with 264 connections (Fig. 4C). In the oviduct-specific network, day 3 and day 18 showed no genes with the maximum FC level of the same tissue.

In summary, the highest number of significant genes was found in the ovary, the lowest number of significant was found in the oviduct (Fig. 4). In addition, we observed different levels of expression between oestrous phases based on the number of genes with maximum FC values through the oestrous phases and three tissues. Peak gene expression was observed between days 6 and 9, whereas the end of the cycle, between days 15 and 18, revealed basal gene expression levels.

Significant pathway terms from the Kyoto Encyclopaedia of Genes and Genomes (KEGG) database were enriched in the list of genes from each of the tissue-specific networks (Fig. 4D–F). The genes with the highest differential expression in the endometrium-specific network showed significant enrichment for eicosanoid metabolism (Fig. 4D), which is related to changes in prostaglandin and inflammation in blood vessels^21^. The genes with the highest differential expression in the ovary-specific network exhibited enrichment in terms related to steroid hormone synthesis (Fig. 4E). Moreover, the ECM-receptor interaction was robustly pronounced under the luteinising hormone-mediated activation in ovarian cancer^22^. In the chart for enriched pathways of the oviduct-specific network showing the genes with the highest differential expression, the ECM-receptor interaction was also significantly enriched (Fig. 4F). The focal adhesion pathway, which has potential roles in embryo transit and placentation, has also been reported^18,23^. The illustrated treemaps displayed significantly enriched GO terms in biological processes (Fig. 4G–I). The highest enrichments for each tissue were: The endometrium-specific genes were broadly associated with GO terms for responses to chemicals and the regulation of multicellular organismal processes (Fig. 4G), whereas the genes in the ovary-specific network were significantly enriched with GO terms for regulation of ion transport and very long-chain fatty acid metabolism (Fig. 4H). The genes in the oviduct-specific network were significantly enriched with GO terms for collagen biosynthesis and response to chemical (Fig. 4I).

### Genes involved in bridging between the tissue-specific sub-networks

**Figure 5:**
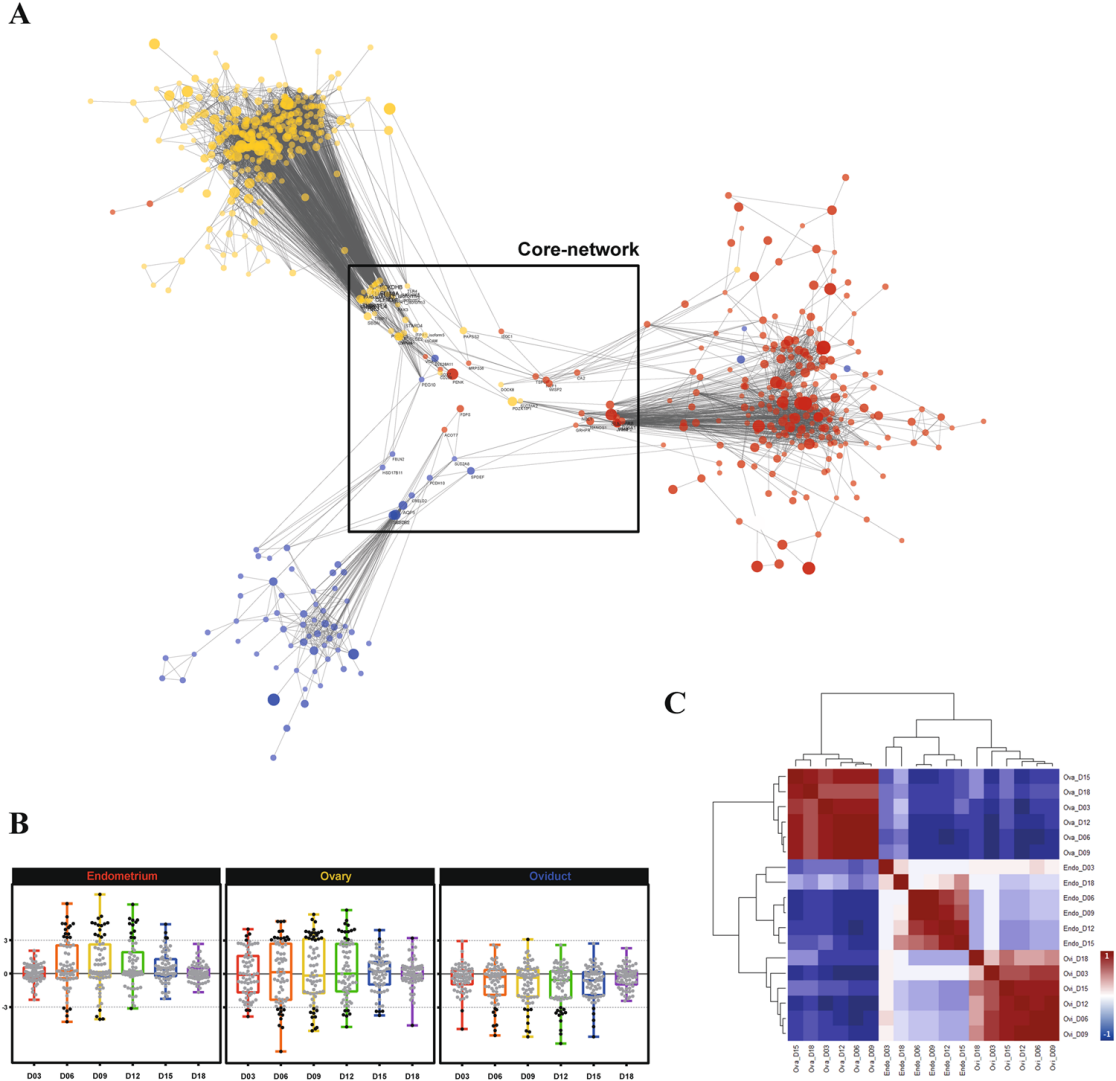
Embedded core network by gene bridging between tissues. (**A**) When a gene (node) has connections (edges) with at least two different tissues (colours), it is designated as a bridging gene. Genes were condensed into a core network, which had a high number of connections with the genes of subnetworks. (**B**) Scattered box plots representing waves of expression in genes of the core network during the oestrous cycle. Black dots in the plots have absolute log2 FC greater than 3.0, while all other dots are grey. The colours of box plots were matched with each of the colours of the oestrous phases in Fig. 4. (**C**) Correlation matrix of the core-network genes. A heat map representing the correlations between genes in different oestrous phases and tissues. The scale of correlation coefficients is coloured with red, white, and blue, representing 1, 0, and −1, respectively.

A network for the entire integrated transcriptome consisted of three robust tissue-specific sub-networks, as shown in Fig. 2. Interestingly, the three sub-networks were connected with a small number of genes forming a triangular shape. Genes that connected at least two sub-networks were designated as bridging genes. In total, there were 65 bridging genes (Supplementary data 3) that had a high level of connectivity (n = 1,454) to all other genes in the network (Fig. 5A). The absolute FC expression levels of genes during the oestrous cycle were highest between day 6 and day 12 in each reproductive tissue (Fig. 5B). As shown in the heat map of the correlation matrix (Fig. 5C), there were many negative correlations between the expression levels in ovary and the other two tissues, whereas there were low correlations between the expression levels in endometrium and oviduct. Moreover, the correlation matrix revealed strong positive correlations in gene expression between day 6 and day 12 in each tissue. Days 3, 15, and 18, however, had relatively low correlations between each other.

The GO enrichment analysis revealed specific clustering for functional annotations of genes. The Table 1 results represented the significantly clustered GO terms such as steroid biosynthetic process and positive regulation of apoptosis that involve in the female reproductive system^24^. The first ranked GO cluster involved biosynthesis of steroid hormones and the following clusters involved cell-to-cell adhesion and cell apoptosis. The GO terms that we identified in the top ranked clusters of reproductive tissues commonly participate in steroidogenesis across the oestrous cycle and possibly regulate reproductive hormone signalling.

**Table 1:** Top five functional GO annotation clusters enriched in categorised GO terms for the core-network genes.

Cluster	Enrichment score	Category^1^	Term	Count	*P*-value	Fold enrichment
1	1.19	BP	steroid metabolic process	4	2.94E-02	5.82
		BP	steroid biosynthetic process	3	3.24E-02	10.38
		BP	lipid biosynthetic process	3	2.92E-01	2.73
2	1.17	MF	monosaccharide binding	3	8.79E-03	20.72
		MF	sugar binding	4	3.16E-02	5.67
		MF	carbohydrate binding	5	3.61E-02	3.90
		BP	cell adhesion	5	2.02E-01	2.10
		BP	biological adhesion	5	2.03E-01	2.10
		BP	cell-cell adhesion	3	2.34E-01	3.20
3	0.90	BP	positive regulation of multicellular organismal process	4	4.72E-02	4.82
		BP	positive regulation of apoptosis	4	1.71E-01	2.74
		BP	positive regulation of programmed cell death	4	1.74E-01	2.72
		BP	positive regulation of cell death	4	1.75E-01	2.70
4	0.83	CC	endoplasmic reticulum	8	5.42E-02	2.27
		CC	endoplasmic reticulum part	4	1.29E-01	3.13
		CC	endoplasmic reticulum membrane	3	2.52E-01	3.03
		CC	nuclear envelope-endoplasmic reticulum network	3	2.72E-01	2.87
5	0.82	CC	proteinaceous extracellular matrix	4	1.08E-01	3.40
		CC	extracellular matrix	4	1.27E-01	3.15
		CC	extracellular region part	6	2.62E-01	1.70

^1^Category for GO terms includes biological process (BP), molecular function (MF), and cellular component (CC).

### qPCR validations for specific target genes

To technically validate genes found in tissue-specific networks, we performed qPCR analysis and compared the expression of those genes determined by qPCR to that by RNA-seq. We have chosen 22 genes included in biological pathways of networks specific for the endometrium, ovary, and oviduct. *HSD11B1*, *FXYD4*, *PTGER*2, *PTGS2*, *CYP2J2*, *SGPP1*, and *SGPP2* were selected for biological pathways in the endometrium-specific network (*aldosterone-regulated sodium reabsorption*, *eicosanoid metabolism*, and *sphingolipid metabolism*), *TLR2*, *TLR4*, *TLR9 AKR1C1*, *CYP7A1*, *CYP17A1*, *CYP19A1*, *ACAT2*, *HMGCR*, *HMGCS1*, *MVK* for biological pathways in the ovary-specific network (*dendritic cells in regulating Th1 and Th2 development*, *steroid biosynthesis*, and *terpenoid backbone biosynthesis*), and *COL1A1*, *COL3A1*, *IGF1*, and *ACTC1* for biological pathways in the ovary-specific network (*focal adhesion* and *hypertrophic cardiomyopathy*).

**Figure 6:**
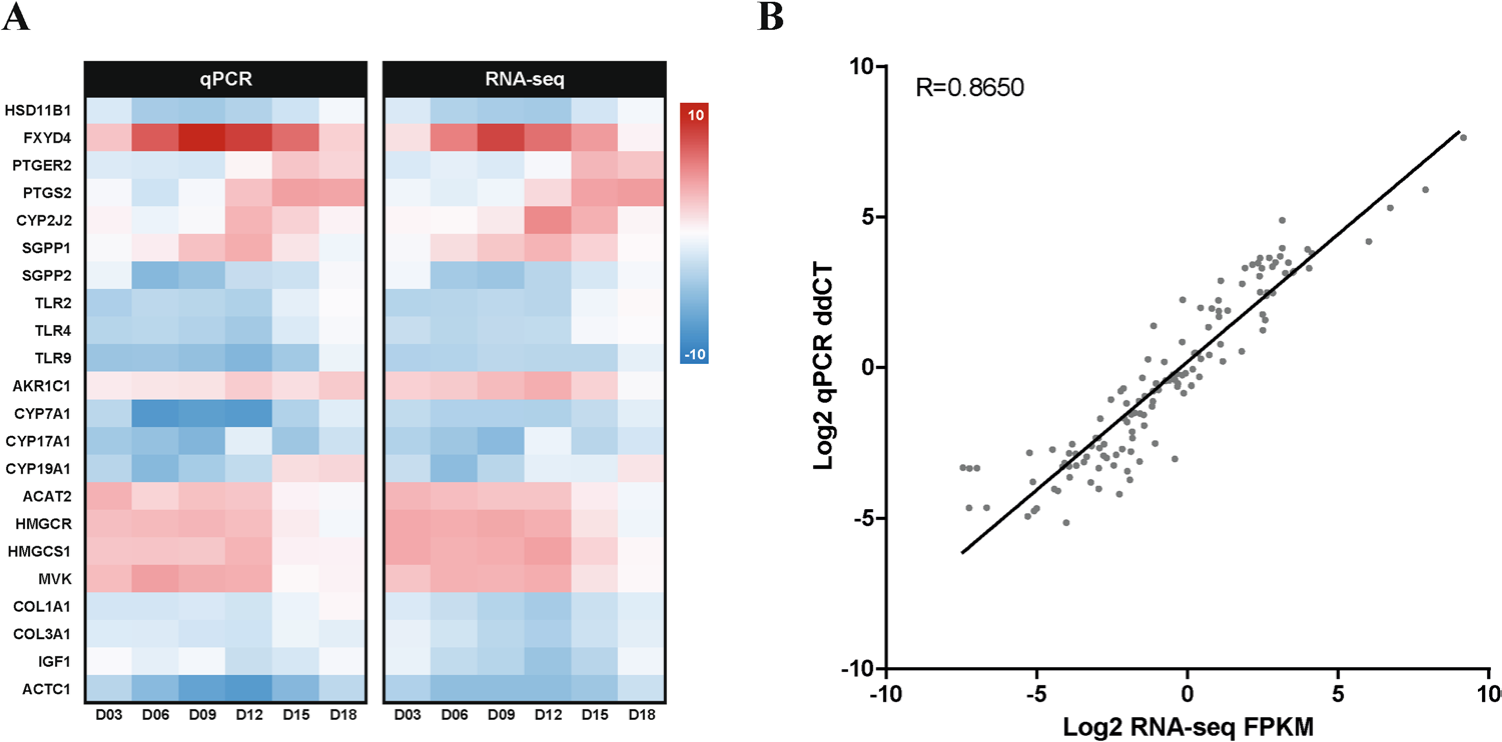
Comparisons of target gene expression by qPCR for technical validation. (**A**) Heatmap representing log2 FC expression values from both qPCR and RNA-seq. The gradient ranges from 10 (red) to −10 (blue). (**B**) Regression plot displaying the direct correlations between the two values for each gene. The seven different time periods of the oestrous cycle are not shown in the plot.

To examine global gene expression during the oestrous cycle between the two experiments, a comparative heatmap was visualized using quantile-normalized values (Fig. 6A). Most gene expression patterns were observed to be analogous between qPCR and RNA-seq throughout the oestrous cycle. To quantitatively assess this observation, the Pearson’s correlation coefficients were calculated using the log-fold change ratio comparison between the two experiments and found a significant correlation in total (*r*^*2*^ = 0.865 and Fig. 6B).

### Localization of genes involved in steroid biosynthesis and eicosanoid metabolism in the reproductive tissues

**Figure 7:**
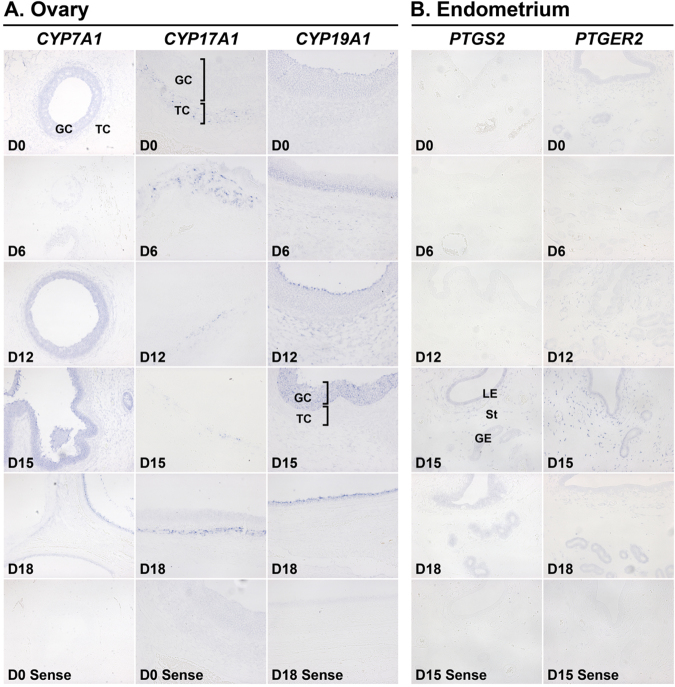
*In situ* hybridization analysis of *CYP7A1*, *CYP17A1* and *CYP19A1* mRNAs in the ovary (**A**) and *PTGS2* and *PTGER2* mRNAs in the uterine endometrium (**B**) during the oestrous cycle in pigs. Representative uterine sections from indicated day of the oestrous cycle hybridized with DIG-labeled sense cDNA probes (Sense) as a negative control are shown for *in situ* hybridization analysis. D, Day; GC, Granulsa cell; TC, Theca cell; LE, luminal epithelium; GE, glandular epithelium; St, stroma. Scale bar = 100 μm.

To understand the cellular localization of the expression of genes found in GCN analysis, we performed *in situ* hybridization analysis. We have chosen *CYP7A1*, *CYP17A1*, and *CYP19A1*, genes included in a biological process, *steroid biosynthesis*, found in the ovary-specific network, and *PTGS2* and *PTGER2*, genes included in a biological process, *eicosanoid metabolism*, found in the endometrium-specific network. As shown in Fig. 7A, the expression of *CYP7A1* and *CYP19A1* mRNAs was detected in the ovarian follicles with strong signal intensity in granulosa cells (GCs), while the expression of *CYP17A* mRNA was detected mainly in ovarian thecal cells (TCs). The expression of endometrial genes, *PTGS2* and *PTGER2*, was localized to endometrial epithelial and stromal cells with strong signal intensity at late diestrus stage of the oestrous cycle (Fig. 7B).

## Discussion

### Differentially expressed genes among tissues and time of oestrus cycle

During the oestrous cycle, the female reproductive system undergoes physiological, morphological, and functional changes in multiple tissues. Hence, understanding the regulatory mechanism of the function of the reproductive tissues is essential for the control of animal productivity and diseases. In this study, we investigated the molecular mechanisms of the oestrous cycle in the female reproductive tissues in pigs. We analysed transcriptomes of the female reproductive tissues of ovary, oviduct and endometrium by comparing and integrating the three transcriptomes throughout the oestrous cycle. According to the PCA plot using the transcriptomes expressed at different time of the oestrous cycle and in different reproductive tissues, a well-defined trajectory of transcriptomes was observed depending on the tissue type. Moreover, the transcriptomes tended to cluster among the days 6, 9, and 12, and among the days 0, 15, and 18 of the oestrous cycle. When the transcriptomes were flared out by DEG values based on day 0, the similarities between tissues could be more clearly displayed. The number of significant DEGs increased until approximately day 9 and then decreased to a steady state. The drift curves of DEGs resemble the curve for progesterone (P4) secretion and can be related to the secretion of prostaglandin (PGF_2α_), which causes a drop in P4 secretion when a female pig undergoes luteolysis^25,26^. However, it was difficult to find similarities between the drift curves for DEGs and oestrogen (E2) or gonadotropins (LH and FSH).

### Dynamic changes during the oestrus cycle within each tissue

According to list of the significant DEGs along the oestrus cycle of the three tissues, we noted the numbers of common and unique DEGs among the three tissues. Upon the release of GnRH and gonadotropins from the hypothalamic–pituitary axis, the oestrous cycle enters the follicular phase which leads to ovulation and the progression to the luteal phase in the ovary^27^. In response to progesterone from the corpus luteum (CL) in the luteal phase, the oviduct and endometrium prepare for pre-implantation embryo development and the establishment for pregnancy, whereas the endometrium initiates the luteolytic process when pregnancy is not established at the end of the luteal phase. Because the ovary, endometrium and oviduct intercommunicate and play major roles in the maintenance of the oestrous cycle, DEGs that are common among the reproductive tissues might be involved in the same biological processes for the oestrous cyclicity in all investigated tissues, while the DEGs that are unique to each of the tissue are likely to be involved in functions specific to the particular reproductive tissue.

The weighted GCN has advantages for representing system-level properties and for identifying specific molecular mechanisms^28–30^. We showed that several integrated transcriptomes can be illustrated at once by GCN analysis using the PCIT algorithm^31^. Only stringent DEGs (absolute FC > 3.0 and FDR < 0.05) were employed for the GCN analysis. The delta-winged network revealed three sub-networks corresponding to each of the reproductive tissues; meanwhile, the sub-networks were connected to each other with a small number of bridging genes. As revealed in the transcriptome integrations and expression pattern clusters in the constructed network, our data clearly showed the dynamic changes along the oestrous cycle in each reproductive tissue. Moreover, the significant nodes in the network displayed tissue-specific sub-networks and the genes were only highly differentially expressed within the tissue-specific sub-networks. This may suggest that the morphological changes during the oestrus cycle within each tissue individually are regulated via differential gene expression. Furthermore, this regulation within each tissue may be related to smaller differential gene expressions regulating tissue synchronization during the oestrus cycle. Dynamic changes in DEGs or significant gene subsets may represent general biological signals. Therefore, the constructed networks were used to investigate the common or specific reproductive mechanisms in the oestrous cycle can reveal specific changes in gene expression.

### The importance of regulation of the steroid hormone and lipid metabolisms

Since the dynamic changes in DEGs of significant gene subsets specify the general biological actions, we constructed gene networks to investigate the common or specific functions of the reproductive tissues during the oestrous cycle. The functional annotation enrichment analysis for KEGG pathways in the constructed gene network revealed many significantly enriched terms. The enriched terms in the ovary-specific sub-network included *terpenoid backbone biosynthesis*, *steroid biosynthesis*, *sterol metabolism*, *cholesterol transport*, and *lipid metabolism*, which are related to steroid hormone biosynthesis and lipid metabolism. In the ovary, steroidogenesis is one of the major activities for sexual and reproductive functions^32^. In response to gonadotrophins, the ovary produces the major steroid hormones, estrogens and progesterone, which play important roles in reproductive tissues as well as a variety of other tissues. It is known that reproduction is directly coupled with lipid metabolism, which is also affected by steroid hormones^33,34^. Furthermore, it has been shown that the DEGs in the ovarian transcriptomes of pigs differing in litter size are involved in steroid biosynthesis, steroidogenesis and lipid metabolism^14^. Thus, the enriched biological terms found in the ovary-specific sub-network in this study indicate that the critical ovarian activities include steroidogenesis and lipid metabolism and that the actions of those DEGs in those biological terms play major roles in the regulation of the oestrous cycle and ovarian function for porcine prolificacy.

In addition, we determined the localization of genes involved in *steroid biosynthesis* found in the ovary-specific network and *eicosanoid metabolism* found in the endometrium-specific network to understand their cellular localization of expression and function in the reproductive tissues. The expression of *CYP7A1* and *CYP19A1* was mainly localized to ovarian GCs and the expression of *CYP17A* was localized to TCs, indicating that the steroidogenic activity in the ovary is mediated by the actions of TC and GC. The mRNA expression levels of *CYP17A1* and *CYP19A1* were great during the proestrus phase. In the endometrium, *PTGS2* and *PTGER2* expression was localized to endometrial epithelial and stromal cells with greatest abundance at late diestrus and proestrus phase, indicating that the endometrial production of PGs is active and PG action on the endometrium may be critical for endometrial function and oestrous cyclicity.

### Immune signalling and regulation of porcine reproduction

There were also significantly enriched KEGG terms in the network that are related to immune signalling, biological oxidation, and the cells and molecules involved in local acute inflammatory response (Fig. 3). The dendritic cell regulation of TH1-TH2 development through antigen-specific T-cell signalling is an important mechanism in the immune response^35^. Eicosanoid metabolism was also significantly enriched in both the main network and the endometrium-specific sub-network (Fig. 4D). Prostaglandins are one of the multiple subfamilies of eicosanoids. They play a major role in inflammation and other oxidative stresses^36,37^. PGF_2α_ secretion can act to lower P4 secretion, which is the signal that pregnancy has failed in pig. These findings demonstrate that the endometrium is directly involved in the biological reaction to a failed pregnancy during the oestrous cycle and involves prostaglandin and P4 signalling^25^. Moreover, a comparison of transcriptomes between pregnant and non-pregnant pigs reveals significant DEGs related to immune processes and pathways^38^, supporting our findings. In humans, after the withdrawal of progesterone induces menstruation, immune and haemostatic factors interact to induce endometrial haemostasis via platelet aggregation, fibrin deposition, and thrombus formation. Consequently, the cessation of menstrual bleeding can be achieved by the control of endometrial haemostasis^39^. Our results suggest that haemostasis and immune signalling are also important modulatory processes in pig and play a functional role in the endometrium during the oestrous cycle in non-pregnant pigs.

### Developmental origin of the three tissues, oestrus cycling and the core network

The oviduct has a developmental origin common to endometrium and is sensitive to changes in ovarian-derived steroid hormones^40^. The oviductal transcriptome in embryo maturation and transport suggested that the oviduct is associated with progesterone signalling and showed that focal adhesions were enriched in the KEGG enrichment analysis^18^. Focal adhesions are integrin receptor-mediated macromolecular complexes that are associated with the extracellular matrix (ECM)^41^. In mammals, the focal adhesions connected with ECM receptors are key mechanisms in pregnancy recognition signalling after oocytes fertilised in the oviduct enter the uterus for gestation. The mechanisms of pregnancy recognition signalling sustain the functional lifespan of the CL, which produces progesterone^21^. Our results with non-pregnant pigs indicated that focal adhesions and ECM-receptor interactions were significantly enriched in the entire network (Fig. 3) as well as the oviduct-specific sub-network (Fig. 4F). The genes in the oviduct-specific sub-network were downregulated throughout the oestrous cycle and their expression pattern was exactly opposite to the pattern of progesterone secretion from the CL. These results suggest that the downregulation of genes in the oviduct is related to the negative response to pregnancy recognition signalling during the oestrous cycle in non-pregnant pigs. Thus, this result may be opposite in pregnant pigs. This may also affect the expression levels of DEGs of the core network connecting the tissue-specific networks. These DEGs may relate to the synchronization of the tissues. Alternatively, pregnancy may induce larger differences due to the different roles of the tissues in pregnancy.

### The core network of bridging genes connecting the transcriptomes of three tissues regulating pig oestrus cycling

Based on the genes bridging among the three reproductive tissues (Fig. 5) and their significant GO terms (Table 1), the connectivity between the tissues and the shared biological roles of the three tissues were investigated. Ovarian steroids and cytokines regulate endometrial gene expression and oviduct function during the oestrous cycle and pregnancy^10,42^. *CYP17A1*, *FDPS*, *HSD17B11*, and *TSPO* were among the genes in the network that are involved in steroid metabolic processes. In particular, *CYP17A1* is known to play a role in steroid hormone synthesis and a recent study that investigated the proteome of the ovine ovary suggested that upregulation of *CYP17A1* is responsible for large litter size^22^. In pigs, *CYP17A1* was also highly expressed in the endometrium during implantation^23^. On the other hand, *CYP17A1* was downregulated, along with *HSD17B11* and *TSPO*, during the oestrous cycle of non-pregnant pigs, as validated by qPCR (Fig. 6A). Moreover, other kinds of *CYP* (cytochrome P450) genes were also significantly downregulated in our study. The core network also revealed significant GO terms for cell adhesions and ECM. *VCAN*, which is a major component of the ECM, was commonly found in the GO terms. *VCAN* activates downstream cytokines such as IL6 and TNF through TLR2 and CD14 and these cytokine changes are important embryo-maternal signals in the porcine female reproductive tract during pregnancy^43–45^. In our study, genes such as the *TLR*s (Fig. 6A) and *VCAN* were significantly downregulated. Therefore, it can be implied that these genes have negative regulatory effects during the oestrous cycle in the reproductive tracts of non-pregnant pigs. We also focused on the high over-expression of *FXDY4* in the endometrium throughout the oestrous cycle (Fig. 6A). A previous study compared transcriptomes during pregnancy and non-pregnancy on day 12 in the endometrium and indicated significantly up- and downregulated DEGs. Among them, *FXDY4* was extremely downregulated during pregnancy [64]. In our study, we showed that *FXDY4* was highly upregulated with the highest expression occurring on day 9. Therefore, the opposite regulation profiles of genes such as CYP17A1 and FXDY4 represented different changes in hormonal and immune signalling of reproductive tissues during pregnancy and non-pregnancy throughout the oestrous cycle. Therefore, we suggest further studies to clarify the molecular functions of these genes so they may be used as molecular markers.

Summarizing, the core network connecting the transcriptomes of the ovary, the oviduct, and the endometrium is enriched for two types of genes: (1) Steroid metabolism probably related to steroid hormone expression regulation, and (2) Communication between cells: ECM, cell adhesion, and immune processes.

## Conclusions

What can we learn from our results about the regulation of tissue synchronization of three reproductive tissues in non-pregnant pigs throughout the oestrous cycle? First of all, the DEGs of the core network showed less differential expression than the DEGs differing among oestrus phases within each tissue. This suggests that the progression through the oestrus cycle in each tissue is regulated through regulation of gene expression. The results suggest that tissue synchronization may either require less regulation – e.g. because it is an intrinsic trait – or that it is regulated outside the core network. Remarkably, the genes of the core network mainly relate to steroid metabolism and regulation of (physical and immune signaling) cellular contacts. This indeed suggest that steroid hormones may be involved, and that direct interactions among cells inducing cellular signalling in each of the tissues are the main regulatory mechanism regulating the events of different tissues during the progression of the oestrus cycle.

## Materials and Methods

### Animals and tissue preparation

All experimental procedures involving animals were conducted in accordance with the Guide for Care and Use of Animals in Research and approved by the Institutional Animal Care and Use Committee of the National Institute of Animal Science (No. 2015-137). Twenty-one crossbred (Landrace × Yorkshire) gilts of similar age (6–8 months) and weight (100–120 kg) were utilised in this study after experiencing at least two oestrous cycles of normal duration (18–22 days). Gilts in the presence of boars were observed daily for oestrous behaviour. The day on which oestrous behaviour was first exhibited was designated as day 0. The ovary, endometrium, and oviduct reproductive tissues of gilts were collected on days 0 (the onset of oestrous behaviour), 3, 6, 9, 12, 15, or 18 of the oestrous cycle (n = 3 gilts/day) by hysterectomy to encompass the whole stage of the oestrous cycle (Fig. 1A). Endometrial tissue was dissected free of myometrium from the middle portion of the uterine horns. Oviductal tissue was collected from the ampulla region and a whole ovary on one side was collected as previously described^46,47^. Tissues were snap-frozen in liquid nitrogen and stored at −80 °C for RNA extraction. For *in situ* hybridisation analysis, cross sections of endometrium, oviduct, and ovary were fixed in 4% paraformaldehyde in PBS (pH 7.4) for 24 h and then embedded in paraffin, as previously described^47^.

### Library preparations and sequencing

Total RNA was extracted from the endometrial, oviductal and ovarian tissues using TRIzol reagent (Invitrogen, Life Technology, Carlsbad, CA) according to the manufacturer’s recommendations. The quantity of RNA was assessed spectrophotometrically, and integrity of RNA was validated following electrophoresis in 1% agarose gel. The mRNA of 1 µg of total RNA was converted into a library of template molecules suitable for subsequent cluster generation using the reagents provided in the Illumina® TruSeq™ RNA Sample Preparation Kit. The first step in the workflow involved purifying the poly‐A containing mRNA molecules using poly‐T oligo‐attached magnetic beads. Following purification, the mRNA was fragmented into small pieces using divalent cations under elevated temperature. The cleaved RNA fragments are copied into first strand cDNA using reverse transcriptase and random primers. This is followed by second strand cDNA synthesis using DNA Polymerase I and RNase H. These cDNA fragments then go through an end repair process, the addition of a single ‘A’ base, and then ligation with adapters. The products were then purified and enriched with PCR to create the final cDNA library. The libraries were quantified using qPCR according to the qPCR Quantification Protocol Guide (Manufacturer) and qualified using an Agilent Technologies 2100 Bioanalyzer. The cDNA libraries were sequenced using the paired-end sequencing by Illumina HiSeq. 2000.

### RNA-seq data processing and differentially expressed gene (DEG) analysis

In total, 130 million paired-end sequence reads were produced, with an average of 19.5 million reads per sample. The all of raw RNA-seq data were deposited at the NCBI Gene Expression Omnibus (https://www.ncbi.nlm.nih.gov/geo/) under the accession GSE108570. Before the RNA-seq data analysis, raw reads were processed for quality filtering by fastQC and the first 13 bp of each read were trimmed based on the Per Base Sequence Content from fastQC^48^. The standard Tuxedo protocol was followed to estimate the normalised expression levels of transcripts^49^. All trimmed reads were mapped against the reference genome (*Sus scrofa* 10.2, GCA_000003025.4) and transcriptome (Ensembl v78) from the Ensembl genome browser (http://www.ensembl.org/Sus_scrofa/) by tophat2 v2.0.13 using the default options. Mapping statuses were plotted and analysed by MultiQC v0.8dev^50^. All possible transcripts were inferred using the Reference Guided Transcriptome Assembly (RABT) mode in cufflinks v2.2.1^51^. The gene expression level was obtained using the measurement of fragments per kilobase per million reads (FPKM). Geometric normalisation methods were used because at least two samples from different pigs were collected for each time point^52^. Differentially expressed genes (DEGs) were selected by Cuffdiff in the Tuxedo protocol with a q-value cut-off of 0.05 by comparing all tissues at the same time point. Principal component analysis (PCA) was performed to analyse relationships between individual samples and we employed ggfortify and cluster packages in R to visualise PCA results. Additional k-means clustering and PAM (Partitioning Around Medoids) algorithms were adopted because individuals were clearly clustered into three groups^53^.

For expression profiling across multiple tissues and periods of the oestrous cycle, further analyses were performed using in-house R scripts. The correlation values between tissues were calculated by Pearson Correlation using a log2 (FPKM + 1) value. Then, the complete-linkage clustering was performed and visualised with an FPKM frequency histogram. Genes that had fold changes (FC) of 2, 4, 8, 16, 32, 64, 128, and 256 were selected and the genes with an 8-FC showing high connectivity among tissues were visualised and analysed^54^. Z-score-transformed log2 (FPKM + 1) values were used to compare and analyse gene expression levels.

### GCN analysis and visualisation

The GCN analysis was performed on transcripts with cut-off proceeded as follows: (1) the transcripts were removed when they did not have a significant FDR (q < 0.05) in any of the seven oestrous time points and three tissues; (2) the rRNA transcripts and *de novo* transcripts that had more than two gene symbols were removed; (3) a stringent significant level in DEG (absolute log2 FC ≥ 3.0) was employed to enhance the efficiency of network construction. As a consequence, only the transcripts that had absolute log2 FC greater than 3.0 in at least one of the seven oestrous time points and one of the three tissues were used for GCN analysis. The significant DEGs were entered in the PCIT algorithm^31^. The PCIT algorithm determined co-expression between genes using the concept of partial correlation coefficients with information theory. Thereafter, the PCIT algorithm has been used to establish significant connections (edges) and construct the network. Connections between two genes with a correlation estimate for co-expression between zero and one. We created a network by taking absolute co-expression correlations greater than 0.90 among those determined to be significant by the PCIT algorithm. Cytoscape version 3.4.0 was used to visualise the co-expression network^55^. In the topological view of the network, genes (nodes) were closer together when they had more common neighbours than the others.

Sub-networks were constructed based on the gene clustering analysis with visual identifications. The k-means clustering algorithm was used in the Multi Experiment Viewer software after median centring by narrowing down the optimal number of clusters with 1 K iterations^56^. The clustered genes had distinct expression patterns between tissues based on their log2 FC of FPKM across all phases of the oestrous cycle. Genes that had connections to at least two different tissues, designated bridging genes, were used to investigate the core-network responsible for connections between tissue-specific networks. Otherwise, genes were excluded when they were embedded within the tissue-specific networks or located outside of sub-networks. Bridging genes were used to rebuild the core network, which was visualised using the Prefuse force directed layout in the Cytoscape software. Then, four sub-clusters were identified that had a high number of connections in the core network. The same colours were always used in the figures to represent the different tissues (red, endometrium; yellow, ovary; and blue, oviduct) and oestrous cycle time points (red, D3; orange, D6; yellow, D9; green, D12; blue, D15; and purple, D18).

### *In silico* functional analyses

The genes in the network were analysed for functional enrichment of Gene Ontology (GO) terms in biological processes and pathways using the DAVID Bioinformatics Resources 6.7^57^. Major databases, including BBID, BIOCARTA, KEGG, PANTHER, and REACTOME, were used for the pathway enrichment analysis. The significantly enriched pathways were represented by fold enrichment and a –log10 *P*-value. GO annotations in biological processes were also enriched using the FAT option, which is a specific GO category filter in DAVID.

The enrichment analysis of pathways in DAVID was performed again for each of the clearly separated tissue-specific sub-networks of different tissues using the same method as described above. GO enrichment analyses for the sub-networks were also carried out by the BiNGO plugin in Cytoscape^58^. Then, the REVIGO visualisation tool was applied to make tree maps for the enriched GO terms^59^. The enrichment analyses using BiNGO were conducted with default conditions and with the basal significance level (*P* < 0.05). The data were analysed using the human annotation (using HUGO official gene symbols) and the pig annotation, separately. The core network, consisting of genes connecting the tissue-specific sub-networks (called “bridging genes”), functional annotation clustering in DAVID was performed to determine the various biological roles of core-network genes in all aspects of GO terms including biological processes, cellular components, and molecular functions.

### Quantitative real-time PCR (qPCR)

The results of RNA-seq analysis were validated by qPCR with the same RNA samples as used for RNA-seq. qPCR was performed with the Applied Biosystems StepOnePlus System (Applied Biosystems, Foster City, CA, USA) using the SYBR Green method. Complementary DNAs (cDNAs) were synthesised from 4 µg of total RNA isolated from the tissues. Newly synthesised cDNAs (total volume of 21 μl) were diluted 1:4 with sterile water and then used for PCR. The Power SYBR Green PCR Master Mix (Applied Biosystems) was used for PCR reactions. The final reaction volume of 20 μl included 2 μl of cDNA, 10 μl of 2 × master mix, 2 μl of each primer, and 4 μl of dH_2_O. PCR conditions and sequences of primer pairs are listed in Supplementary data 1. The cycle thresholds determined for the target genes were normalised against the geometric mean of the reference genes, *RPL7*, *UBB*, and *TBP*, which have previously been used in porcine reproductive tissues^47,60^. Relative expression levels of the selected target genes were calculated using the 2^−ΔΔCT^ method^61^. The identity of each amplified PCR product was verified by sequence analysis after cloning into the pCRII vector (Invitrogen). Regression analysis was performed to compare the expression values from the real-time PCR with RNA-seq results.

### Non-radioactive *in situ* hybridisation

To determine the localisation of expression of selected genes in the reproductive tissues, a non-radioactive *in situ* hybridisation procedure was performed, as previously described with several modifications^62^. Sections (5 µm thick) were rehydrated through successive baths of xylene, 100% ethanol, 95% ethanol, diethylpyrocarbonate (DEPC)-treated water, and DEPC-treated PBS. Tissue sections were boiled in citrate buffer (pH 6.0) for 10 min. After washing in DEPC-treated PBS, sections were digested using 5 µg/ml Proteinase K (Sigma) in TE (100 mM Tris-HCl, 50 mM EDTA, pH 7.5) for 10 min at 37 °C. After post-fixation in 4% paraformaldehyde, tissue sections were incubated twice for 15 min each in PBS containing 0.1% active DEPC, and equilibrated for 15 min in 5 × saline sodium citrate (SSC). The sections were prehybridised for 2 h at 68 °C in hybridisation mix (50% formamide, 5 × SSC, 500 µg/ml herring sperm DNA, 250 µg/ml yeast tRNA). Sense and antisense riboprobes for each gene were generated using partial cDNAs cloned into pCRII vectors by linearising with appropriate restriction enzymes and labelling with digoxigenin (DIG)-UTP using a DIG RNA labelling kit (Roche, Indianapolis, IN, USA). The probes were denatured for 5 min at 80 °C and added to the hybridisation mix. The hybridisation reaction was carried out overnight at 68 °C. Sections hybridised with sense riboprobes served as negative controls. Pre-hybridisation and hybridisation reactions were performed in a box saturated with a 5 × SSC − 50% formamide solution to avoid evaporation. No coverslips were used. After hybridisation, sections were washed for 30 min in 2 × SSC at room temperature, 1 h in 2 × SSC at 65 °C, and 1 h in 0.1 × SSC at 65 °C. Probes bound to the section were detected immunologically using sheep anti-DIG Fab fragments covalently coupled to alkaline phosphatase and nitro blue tetrazolium chloride/5-bromo-4-chloro-3-indolyl phosphate (toluidine salt) as chromogenic substrate, in accordance with the manufacturer’s protocol (Roche).

## Electronic supplementary material

Supplementary information
