# Supplementary material for: Integrated transcriptomes throughout swine oestrous cycle reveal dynamic changes in reproductive tissues interacting networks
Source: Sci Rep. 2018 Apr 3;8:5436. doi: 10.1038/s41598-018-23655-1 (PMC5882957; doi:10.1038/s41598-018-23655-1)
Supplement: Supplementary file 1 — Supplementary information [file 41598_2018_23655_MOESM1_ESM.pdf]

Integrated transcriptomes throughout swine oestrous cycle reveal dynamic changes in reproductive tissues interacting networks

Jun-Mo Kim, Jong-Eun Park, Inkyu Yoo, Jisoo Han, Namshin Kim, Won-Jun Lim, Eun-Seok Cho, Bonghwan Choi, Sunho Choi, Tae-Hun Kim, Marinus F. W. te Pas, Hakhyun Ka, and Kyung-Tai Lee

Supplementary data 1. Summary of qPCR primer sequences and expected product sizes

Primer	Sequence of forward (F) and reverse (R) primers (5' → 3')	Annealing Temperature (°C)	Product size (bp)	No. of cycles	GenBank accession no.
ACAT2	F: CTACCACATGGGCATCACAG<br>R: ACAAAAACCGGCACAATCTC	60	150	40	XM_001928345.3
ACTC1	F: TGCTGAGCGTGAAATTGTTC<br>R: ATGCCAGCAGATTCCATACC	60	213	40	NM_001170517.2
AKR1C1	F: TTTTCCGTCCAGAATTGGTC<br>R: CCTCGTTCTTGCACTTCTCC	60	207	40	NM_001044618.1
COL1A1	F: CAAAGAAGGCAGCAAAGGTC<br>R: TCCAGCAATACCCTGAGGTC	60	163	40	XM_013981012.1
COL3A1	F: TTTGGCACAACAACAGGAAG<br>R: TCTCCAAATGGGATCTCAGG	60	189	40	NM_001243297.1
CYP2J2	F: GCCTCCAGACAATGAGAAGC<br>R: CTGTCCCCTCACTTCTGAGC	60	180	40	XM_003127961.5
CYP7A1	F: TGCACAGAAAGCACTCATCC<br>R: ACCAGTTCCGAGATGTGGTC	60	183	40	KP687249.1
CYP17A1	F: CCAAGGAGGTGCTTCTCAAG<br>R: GTTCTCCAGCTTCAGGTTGC	60	188	40	NM_214428.1
CYP19A1	F: TGCTAATTGCAGCACCAGAC<br>R: GGCTGGTACCTCATGCTCTC	60	196	40	NM_214429.1
FXYD4	F: AACTGTGTCATGGAGGGTGT<br>R: ATGGAAACTGGTACAGGGAG	60	318	40	XM_013983257.1
HMGCR	F: GCAGATGCTAGGGGTTCAAG<br>R: GTGCAAGTTCCTTGGAGGTC	60	198	40	EU726797.1
HMGCS1	F: GCGGCTAGAAGTTGGAACAG<br>R: ACAGCAATATCCCCTGCAAC	60	222	40	NM_001252215.1
HSD11B1	F: AAGAAGGTGGTGTCCCACTG<br>R: GGACCACGTAGCTGAGGAAG	60	235	40	KP735788.1
IGF1	F: CTCTCCTTCACCAGCTCTGC<br>R: CTCCAGCCTCCTCAGATCAC	60	201	40	NM_214256.1
MVK	F: TGGAGCATTACCTTGTGCTG<br>R: GCCTGCTTCGTGTCCTCTAC	60	214	40	XM_001929184.5
MYH7	F: CTACTCGGGCCTCTTCTGTG<br>R: ACTGGATGACCCTCTTGGTG	60	233	40	NM_213855.1
PTGER2	F: AGAGTAGGCTTGGTTGGTTAATGTC	60	129	40	XM_003353488.3

	R: GAAACTTCCTAACGTCTTCTTGGTT				
PTGS2	F: TCGACCAGAGCAGAGAGATGAGAT	60	134	40	NM_214321.1
	R: ACCATAGAGCGCTTCTAACTCTGC				
SGPP1	F: TCTGGATCTGGAACCTGGAC	60	222	40	XM_003353501.3
	R: AGGTGAGCAGAACCATGGAG				
SGPP2	F: TCCTTGGTGTGTCTCAGCAG	60	243	40	XM_013984536.1
	R: CAGACACCACCACCATTCTG				
TLR2	F: GAACCCATCGAGAAGAAGACCATTC	60	148	40	NM_213761.1
	R: GAACCTAGGACTTGATCGCAGCTCT				
TLR4	F: TAGTGCGTCAGTTCTCACCTTCCTC	60	119	40	AB188301.2
	R: AGGTGCTTTCACCTCTGCCATATTT				
TLR9	F: GCCATTACTAGGGAGGTGGATGGT	60	109	40	NM_213958.1
	R: TGCAGTTTGGCATGAAGTCCTCT				
RPL7	F: AAG CCA AGC ACT ATC ACA AGG AAT ACA	60	172	40	NM_001113217
	R: TGC AAC ACC TTT CTG ACC TTT GG				
UBB	F: GCA TTG TTG GCG GTT TCG	60	65	40	NM_001105309.1
	R: AGA CGC TGT GAA GCC AAT CA				
TBP	F: AAC AGT TCA GTA GTT ATG AGC CAG A	60	153	40	XM_013991786.1
	R: AGA TGT TCT CAA ACG CTT CG				

Supplementary data 2. Gene clusters based on expression pattern clustering

Clusters	Number of genes (%)	Gene symbol
Cluster 1	58 (9 %)	MUC20, UABP-2, NOS1, RAI2, CHGA, CYP24A1, SOD3, KSR2, MMP7, CLU, WISP2, BF, LAMB3, CRYGS, MUC16, SLPI, LCN2, LGR5, UABP-2, CXCL2, LAMC2, VNN2, HSD11B1, UCAL-P19, CHIA, SELL, PLAT, SULT2A1, SLC38A4, UPK1B, RASD1, GPX3, MYO10, TIMP-2, MEP1B, BCAS1, C2CD4A, PTGER2, SLC47A2, SGPP2, SGPP2, MUC4, HTRA4, COL17A1, IER3, C2orf72, LIF, COL17A1, VNN1, UNC5CL, PRKAR2B, IGFBP3, PVALB, SLC12A2, GCNT3, CA2, PTGS2, IL1A
Cluster 2	136 (22 %)	KCNK2, HAO2, GRAMD1B, HIST1H2BK, GDAP1L1, LIMA1, LIMA1, LHCGR, ITPR1, PAQR9, L1CAM, S1PR5, RBMS3, ODC1, HUNK, SYT3, RGC32, MAP1B, PPP1R1B, PLS1, UBR3, OLFM3, CYB5A, CLIC6, SMARCA1, DUOXA1, FMO1, TMPRSS7, KCNE1, MEDAG, ENPEP, CD209, SLC30A5, FAM195A, WFIKKN1, BSP1, SLC26A6, GGT1, TGM5, FOXRED2, DEFB123, PNCK, TGFA, ITPR1, SULT1C3, CLIC5, TDH, DKK2, TMEM170B, COBLL1, IL4I1, DUOXA2, ABCB1, MAL2, OCRL, AP1S3, COBL, CACNA2D2, PCDH15, ERO1B, SNTB1, CYB561, DUOX2, LHCGR, PEG10, ITPR1, ITPR1, ITPR1, KCNK3, PAK3, PAK3, PIM2, TM7SF2, PRKX, FDXR, JAKMIP1, FAM222A, PCOLCE2, HAGH, DNAJA1, HAGH, SOX7, RDH11, SCAP, GAS2, GLDC, RUNDC3A, SLC35F5, AKR1C1, FAM196A, B3GALNT1, PGP1A, HUNK, AVPI1, AQP9, VAT1L, ERO1B, UGT8, BCKDHB, ELOVL6, SOHLH1, RLN, PTHLH, MVK, CCDC64, TMEM52, COL22A1, DHRS4, DHCR24, EBP, ADRB1, RASGRP1, AOX1, ST8SIA5, MME, FABP, FABP3, FAM101A, MRO, DERL3, CTAGE4, AIG1, OLFM2, SLC38A11, ABCB1, RDH12, ALOX15, FSTL4, IBSP, SORD, UGT8, SLC5A2, VCAN, TIMP1, PTGFR, PDZK1IP1
Cluster 3	81 (13 %)	EPHB1, TP53INP2, FAXDC2, LIPG, RNF152, C1QL4, EV15, OATV1, FBXO2, SLC2A8, SPDEF, ADAMTS12, ADRA1D, CA12, WNT2, CRELD2, C16orf89, FDPS, CREB3L4, ACOT7, SGPP1, PEBP4, PCDH10, PAPSS2, MRPS36,

		HAPLN3, RCAN2, ACTC1, AQP5, MYH13, BTBD17, SDF2L1, BRINP1, LBP, BRINP1, FAM3B, ACTA1, CENPW, LMAN1L, GRM4, MFSD2A, RAB27B, CA3, MYH7, SYNDIG1, COL1A1, COL1A1, COL1A1, KCNG1, AGTR2, FAM3B, SAA1, CNIH2, ZNF385C, CASQ1, COL1A2, TNNI1, C1QTNF6, GPC3, OLFM4, CKM, FBLN2, HMGCS1, REXO2, PLA2G3, SC4MOL, CPXM1, ST8SIA5, CITED1, HMGCR, PANK3, SAA2, ACLY, ANXA13, ACAT2, FBLN2, HSD17B11, UBTFL1, BCKDHB, STARD4, COL3A1
Cluster 4	183 (29 %)	CH242-277I8.1, IGKC, HSPB3, HK3, TNC, VGF, TNC, CXCL14, INSL3, CYP17A1, IGLV-3, GCG, PTPRN, CYP7A1, ZP3, CDKN2B, ANKRD22, DCLK1, DCLK1, SLC6A4, NTRK1, TNFRSF9, TCN1, TRPM2, STRA6, FKBP6, EMR4, NCCRP1, DOCK8, TSLP, FAM134B, SCG2, MEST, OLR1, CH242-305H5.1, WDFY4, CSF3R, PADI2, FNDC1, DOK2, EVA1A, SEMA4A, ITGAX, MERTK, ADCY7, CYP19A1, ISLR2, SBSN, PYCARD, CTSL, APRIL, TREM2, TCN1, CLEC5A, SMPDL3A, CD36, TLR9, SV2C, OLFM1, SKM, CD37, ITGAM, GAREML, TREH, RASGRP4, ZAR1, C19orf38, CH242-168I5.2, IL33, SDC4, ENPP1, FN1, ST14, F2RL1, SLC2A5, FCN1, CLEC7A, CTHRC1, SLC11A1, ADCY7, CD68, LAT2, MCF2L, SCARNA13, LRP4, TREM2, APOE, LPXN, MAFB, AMBN, PILRA, IL2RG, PCD1A, LCP1, BCL2A1, ERVV-2, TMEM86A, GIMAP8, PTPRCAP, C5AR1, LPXN, NKG7, ARL11, TRPV2, C3AR1, TNFRSF8, LAIR1, NCKAP1, SPN, WNT5B, PDK4, VAV1, VSIG4, ITGB2, SLA, SPI1, MYO1G, ADRB2, GIMAP6, SLA-DQA1, MAP4K1, CD48, GIMAP1, LPPR3, C16orf54, ARHGAP30, P2Y12R, C3, MMP13, C3, CSF2RA, CECR1, PLD4, FGD2, TUBB3, DOCK2, GIMAP4, CASP1, SLA-DRB, GNA15, HCK, NCKAP1, RGS10, TLR2, RASAL3, RASAL3, LYZ, MYO1F, KMO, PARVG, FCER1G, MPEG1, CTSS, CSF2RB, VSIG4, GMFG, CD53, NCF2, HNMT, TYROBP, ADAM28, SLA-DMB, SOX4, APOD, CADM1, LCP2, SRGN, GPX6, UPK3B, CX3CR1, SNCG, DF, MSR1, COL28A1, ABCC4, SELPLG, TLR4, SCRG1, C14H10orf116, ZC3H12D, STRIP2, IGLV-8, DPPA5
Cluster 5	164 (26 %)	PBD-2, NDP, CHRDL1, COBL, PI15, PPAPDC1A, FAM169B, CYP2J2, PGC, TRPV5, NBL1, SLC23A1, UPTI, SMOC1, SLC24A4, SYT13, CLDN8, NMU, ELOVL2, NANOS1, SOWAHA, SLC28A2, GDA, CH242-204P3.6, COBL,

	PROB1, DKK1, FGF7, CREB, CD69, ZFYVE28, COBL, EGF, IFN-EPSILON, GPER1, SLC9A4, CYB5R1, FAM169B, BTNL9, CYP2J2, MMP8, CHRFAM7A, SLC28A3, PCDHAC2, IL24, ST6GALNAC1, JPH4, MOCOS, SLC5A1, WWP2, CC2D2B, MOCOS, SCNN1A, IGLV-7, SLC31A2, CES3, IL17REL, CCK, ANKRD34B, OPTC, SV2C, ENTPD1, FXYD4, SLC27A6, SLC18A2, CYFIP2, KCNE3, TFCP2L1, IGKV-7, IGLV-9, GRIA1, GRIA1, RNF223, TCTN3, RLBP1, DGKI, CERS6, ADRA2C, PENK, GRIA1, OAT, HPGD, CWH43, NCF1, TSPO, SPINK2, CLDN22, SLC16A6, RNF122, MAST2, GPR110, CDH17, GLYATL2, GALNT18, SORL1, KIAA1191, ATP13A4, MOGAT1, CEMIP, CHL1, GABRP, SMPD3, IGSF1, CA8, ENPP3, KIAA1324L, VPS37A, SLCO1A2, TMEM88B, CCT6B, KLRG2, SLC3A1, OGDH, DOCK9, MPP7, ENPP3, NABC1, CCL28, GREB1L, DGKG, DGKG, DGKG, FRMD3, ATP8A1, PHGDH, SLC6A15, TMEM200B, JPH3, CILP2, HK2, SLC4A11, PHGDH, THEM5, GPR183, GPIBA, CHODL, SLC7A3, SLC26A4, CYP26A1, RPRML, TMC5, XDH, GSTA4, RFK, SLC7A11, GPHA2, LRRN1, CLCN4, SLC4A11, RHOBTB3, PDZK1, GLYATL, SULT2B1, TMEM63C, DNMT3B, MYRF, GRHPR, DNER, GPT2, GAT, CDH16, ELFN2, IGF1, ISOC1

Supplementary data 3. List of differential expressed genes for entire of network and each of sub-networks

Category	Number of genes	Gene symbol
Entire network	622	WNT2, PLAT, TREH, PILRA, TNC, SCRG1, RASGRP4, IER3, CRELD2, ZAR1, IL2RG, C19orf38, CD209, KCNK2, CPXM1, SLC30A5, GPX6, GRM4, C16orf89, UPK3B, SLC31A2, CX3CR1, CHGA, MFSD2A, RAB27B, SULT2A1, CH242-168I5.2, ST8SIA5, FAXDC2, FAM195A, CDKN2B, WFIKKN1, IL33, FDPS, HAO2, SDC4, CREB3L4, BSP1, ANKRD22, IGF1, GRAMD1B, ACOT7, SLC26A6, SGPP1, P2Y12R, GGT1, TGM5, FOXRED2, NBL1, C3AR1, SAA1, SLC23A1, PEBP4, MUC20, DEFB123, DCLK1, SNCG, GALNT18, PNCK, GRHPR, CES3, GRIA1, GRIA1, PBD-2, C3, TGFA, WWP2, UPTI, SLC38A4, LCN2, ITPR1, GSTA4, RFK, UPK1B, SULT1C3, IL17REL, CCK, C1QTNF6, ANKRD34B, CA3, CCT6B, MYH7, LGR5, C14H10orf116, SLC7A11, RNF223, TCTN3, SYNDIG1, DCLK1, PCDH10, SORL1, RASD1, KLRG2, KIAA1191, ATP13A4, SLC3A1, GPHA2, SLC6A4, NTRK1, CLIC5, TDH, GPC3, GPX3, C2orf72, MYO10, TNFRSF9, MMP13, C3, TNFRSF8, TCN1, CSF2RA, PCD1A, DKK2, CASQ1, SMOC1, CNIH2, SLC24A4, SYT13, TRPM2, TMEM170B, ST6GALNAC1, COBLL1, IL4I1, DUOXA2, CLDN8, ABCB1, HIST1H2BK, MAL2, OCRL, GDAP1L1, AP1S3, COBL, LIMA1, LIMA1, CACNA2D2, CITED1, CYP24A1, HMGCR, RLBP1, NMU, OGDH, ELOVL2, ENPP1, STRA6, LRRN1, SLC12A2, STARD4, PAPSS2, MRPS36, LIF, FKBP6, COL17A1, EMR4, GCNT3, NCF1, VNN1, DGKI, LAIR1, CA2, TSPO, PANK3, SPINK2, UNC5CL, HAPLN3, NCCRP1, CERS6, LIPG, RNF152, PCDH15, ISOC1, C1QL4, RCAN2, DOCK8, TIMP-2, UABP-2, ERO1B, MEP1B, UABP-2, SNTB1, SOD3, KSR2, JPH4, OPTC, NDP, NANOS1, MOGAT1, BCAS1, NCKAP1, SOWAHA, FN1, CHRDL1, CYB561, SLC28A2, COBL, DUOX2, TSLP, FAM134B, SCG2, LHCGR, VGF, LHCGR, TNC, CEMIP, C2CD4A, OLFM4, MEST, PTGS2, PTGER2, MOCOS, GDA, CH242-204P3.6, PI15, PCDHAC2, CRYGS, CLCN4,

	COBL, ITPR1, PROB1, PEG10, PPAPDC1A, DOCK9, ITPR1, HSD11B1, ADRA2C, ITPR1, ITPR1, MPP7, ENPP3, KCNK3, PAK3, OLR1, PAK3, CH242-305H5.1, DNER, WDFY4, DKK1, PIM2, PRKAR2B, SLC4A11, ST14, COL1A1, F2RL1, COL1A1, CSF3R, FGF7, COL1A1, SLC2A5, IL1A, COL1A2, FCN1, TM7SF2, PAQR9, KCNG1, PRKX, LCP1, FDXR, JAKMIP1, PADI2, FAM222A, PENK, FNDC1, DOK2, PCOLCE2, EVA1A, HAGH, DPPA5, DNAJA1, SEMA4A, CLEC7A, PDZK1IP1, HAGH, IGFBP3, ITGAX, SOX7, MERTK, L1CAM, RDH11, SCAP, BCL2A1, CTHRC1, SAA2, GAS2, CKM, GLDC, DF, COL3A1, NABC1, RUNDC3A, ADCY7, CYP19A1, S1PR5, SLC35F5, CECR1, ISLR2, PLD4, SLC11A1, CH242-277I8.1, FGD2, AKR1C1, RBMS3, GPT2, ERVV-2, TUBB3, FAM196A, MMP7, MSR1, CCL28, DOCK2, RHOBTB3, GREB1L, DGKG, B3GALNT1, CXCL2, DGKG, DGKG, ODC1, GIMAP4, PGP1A, CASP1, HUNK, HUNK, TMEM86A, AVPI1, SYT3, AQP9, RGC32, MAP1B, TNNI1, GIMAP8, ACTC1, VAT1L, AGTR2, AQP5, SBSN, MYH13, BTBD17, SDF2L1, ERO1B, ZNF385C, UGT8, COL28A1, BRINP1, EV15, OATV1, PYCARD, LBP, FRMD3, ABCC4, CTSL, BRINP1, FAM3B, FAM3B, CHL1, UCAL-P19, SLA-DRB, MUC16, SELPLG, GRIA1, ATP8A1, GNA15, LAMB3, HCK, PDZK1, GABRP, NCKAP1, RGS10, SMPD3, TLR2, PHGDH, CLDN22, ACTA1, CENPW, SLC6A15, CLU, SLC47A2, TMEM200B, OAT, JPH3, IGSF1, SGPP2, CILP2, SLC16A6, HK2, CHIA, SLC4A11, SELL, PHGDH, GLYATL, CA8, ACLY, ENPP3, CREB, GAT, KIAA1324L, VPS37A, LAMC2, SLCO1A2, ZC3H12D, CD69, ZFYVE28, BCKDHB, COBL, EGF, IFN-EPSILON, THEM5, RASAL3, CC2D2B, RASAL3, IL24, SGPP2, PPP1R1B, GPER1, LYZ, SLC9A4, ELOVL6, CYB5R1, GPR183, GPIBA, SLPI, CHODL, MUC4, SPN, CXCL14, FAM169B, MYO1F, FAM169B, ANXA13, NOS1, KMO, PARVG, WNT5B, INSL3, SULT2B1, FCER1G, SOHLH1, PDK4, MPEG1, BTNL9, RLN, CTSS, CYP2J2, PLS1, MMP8, SV2C, CYP2J2, VAV1, CHRFAM7A, PGC, ACAT2, CSF2RB, VSIG4, RNF122, PTHLH, VSIG4, FBLN2, FBLN2, MAST2, SLC7A3, GMFG, MVK, CD53, FBXO2, ITGB2, NCF2, SLA, SPI1, SLC2A8, SPDEF, ADAMTS12, MYO1G, WISP2, ADRA1D, BF, RAI2,

		CCDC64, HNMT, TMEM52, HTRA4, TYROBP, TLR4, ADAM28, COL22A1, SLA-DMB, SOX4, DHRS4, ADRB2, GIMAP6, APOD, DHCR24, CADM1, UBR3, CDH16, ELFN2, SLA-DQA1, EBP, ADRB1, ADCY7, LCP2, OLFM3, PTPRCAP, CD68, MAP4K1, LAT2, RASGRP1, CD48, AOX1, GIMAP1, MCF2L, SRGN, APRIL, SCARNA13, ST8SIA5, HPGD, LPPR3, HMGCS1, ENTPD1, MME, FABP, FABP3, FXYD4, MOCOS, PVALB, GPR110, SLC27A6, SLC5A1, REXO2, FAM101A, CDH17, HSD17B11, MRO, DERL3, SLC18A2, SCNN1A, CWH43, COL17A1, SLC28A3, CYFIP2, KCNE3, CYB5A, TRPV5, CTAGE4, AIG1, OLFM2, CLIC6, SMARCA1, SLC38A11, DUOXA1, ABCB1, RDH12, TFCP2L1, TMEM63C, DNMT3B, SLC26A4, IGLV-7, CYP26A1, IGKV-7, PLA2G3, GLYATL2, IGKC, UBTFL1, RPRML, IGLV-8, IGLV-9, TMC5, XDH, TMEM88B, CYP17A1, MYRF, ALOX15, VNN2, LRP4, FMO1, TREM2, TCN1, TREM2, CLEC5A, C16orf54, FSTL4, SC4MOL, SMPDL3A, CD36, C5AR1, APOE, LPXN, TLR9, IGLV-3, IBSP, HSPB3, GCG, PTPRN, TMPRSS7, SV2C, LPXN, MAFB, OLFM1, ARHGAP30, NKG7, CYP7A1, ARL11, ZP3, SORD, SKM, EPHB1, STRIP2, UGT8, SLC5A2, BCKDHB, LMAN1L, KCNE1, VCAN, TP53INP2, TRPV2, TIMP1, HK3, MEDAG, PTGFR
Sub-network (Endometrium)	216	DNER, PLAT, DKK1, PRKAR2B, SLC4A11, IER3, FGF7, IL1A, PENK, SOX7, NABC1, CHGA, GPT2, MMP7, SULT2A1, CCL28, RHOBTB3, GREB1L, DGKG, FAXDC2, CXCL2, DGKG, DGKG, SGPP1, NBL1, SLC23A1, MUC20, FRMD3, CHL1, UCAL-P19, GALNT18, GRHPR, MUC16, GRIA1, CES3, ATP8A1, LAMB3, PDZK1, GRIA1, GABRP, GRIA1, SMPD3, PBD-2, PHGDH, CLDN22, SLC6A15, CLU, SLC47A2, TMEM200B, OAT, JPH3, WWP2, IGSF1, SGPP2, CILP2, SLC16A6, UPTI, SLC38A4, HK2, CHIA, SLC4A11, SELL, PHGDH, GLYATL, CA8, LCN2, GSTA4, ENPP3, RFK, CREB, GAT, UPK1B, KIAA1324L, VPS37A, LAMC2, SLCO1A2, IL17REL, ZFYVE28, COBL, EGF, CCK, IFN-EPSILON, THEM5, CC2D2B, ANKRD34B, IL24, CCT6B, SGPP2, GPER1, SLC9A4, CYB5R1, LGR5, GPR183, GPIBA, SLPI, CHODL, MUC4, FAM169B, SLC7A11, FAM169B, RNF223, NOS1, TCTN3, SULT2B1,

		SORL1, RASD1, KLRG2, BTNL9, KIAA1191, CYP2J2, ATP13A4, MMP8, CYP2J2, SLC3A1, GPHA2, CHRFAM7A, PGC, RNF122, GPX3, C2orf72, MYO10, MAST2, SLC7A3, WISP2, ADRA1D, SMOC1, BF, SLC24A4, SYT13, RAI2, HTRA4, ST6GALNAC1, CLDN8, HPGD, CYP24A1, ENTPD1, RLBP1, FXYD4, MOCOS, PVALB, NMU, OGDH, GPR110, ELOVL2, SLC27A6, SLC5A1, CDH17, LRRN1, SLC18A2, SCNN1A, CWH43, COL17A1, SLC28A3, CYFIP2, KCNE3, MRPS36, TRPV5, CTAGE4, COL17A1, GCNT3, NCF1, VNN1, DGKI, CA2, TSPO, TFCP2L1, SPINK2, UNC5CL, TMEM63C, DNMT3B, SLC26A4, CYP26A1, CERS6, GLYATL2, RPRML, RNF152, TMC5, ISOC1, XDH, TIMP-2, TMEM88B, MYRF, VNN2, UABP-2, MEP1B, UABP-2, SOD3, KSR2, JPH4, OPTC, NDP, NANOS1, MOGAT1, BCAS1, SOWAHA, CHRDL1, SLC28A2, COBL, C2CD4A, PTGS2, PTGER2, MOCOS, GDA, CH242-204P3.6, PI15, PCDHAC2, CRYGS, CLCN4, COBL, PROB1, PPAPDC1A, DOCK9, HSD11B1, ADRA2C, MPP7, ENPP3
Sub-network (Ovary)	324	CD37, KCNK3, AMBN, PAK3, OLR1, ENPEP, ITGAM, PAK3, CH242-305H5.1, GAREML, WDFY4, TREH, PIM2, PILRA, ST14, TNC, F2RL1, RASGRP4, CSF3R, SLC2A5, FCN1, TM7SF2, ZAR1, PAQR9, PRKX, LCP1, IL2RG, FDXR, JAKMIP1, PADI2, FAM222A, C19orf38, CD209, FNDC1, DOK2, PCOLCE2, EVA1A, HAGH, DPPA5, KCNK2, DNAJA1, SLC30A5, SEMA4A, CLEC7A, PDZK1IP1, HAGH, IGFBP3, ITGAX, MERTK, L1CAM, RDH11, SCAP, BCL2A1, SLC31A2, CTHRC1, GAS2, GLDC, DF, CX3CR1, RUNDC3A, ADCY7, CYP19A1, S1PR5, SLC35F5, CECR1, ISLR2, PLD4, SLC11A1, CH242-277I8.1, FGD2, AKR1C1, RBMS3, ERVV-2, TUBB3, FAM196A, RAB27B, MSR1, CH242-168I5.2, DOCK2, ST8SIA5, B3GALNT1, FAM195A, ODC1, CDKN2B, GIMAP4, PGP1A, WFIKKN1, CASP1, HUNK, IL33, HAO2, HUNK, SDC4, BSP1, ANKRD22, TMEM86A, AVPI1, SYT3, GRAMD1B, AQP9, SLC26A6, RGC32, MAP1B, P2Y12R, GGT1, GIMAP8, TGM5, FOXRED2, VAT1L, C3AR1, SBSN, ERO1B, UGT8, PYCARD, CTSL, DEFB123, DCLK1, PNCK, SLA-DRB, SELPLG, GNA15, HCK, NCKAP1, RGS10, TLR2, C3, TGFA, ITPR1, ACLY, SULT1C3, BCKDHB, RASAL3, RASAL3, PPP1R1B,

		LYZ, ELOVL6, SPN, CXCL14, MYO1F, KMO, PARVG, WNT5B, INSL3, FCER1G, SOHLH1, PDK4, DCLK1, MPEG1, RLN, CTSS, PLS1, SV2C, VAV1, SLC6A4, ACAT2, CSF2RB, NTRK1, CLIC5, TDH, VSIG4, PTHLH, VSIG4, TNFRSF9, GMFG, MMP13, MVK, C3, CD53, TNFRSF8, ITGB2, TCN1, CSF2RA, NCF2, SLA, SPI1, MYO1G, PCD1A, DKK2, TRPM2, CCDC64, HNMT, TMEM170B, TMEM52, TYROBP, TLR4, ADAM28, COL22A1, SLA-DMB, SOX4, DHRS4, ADRB2, GIMAP6, APOD, DHCR24, CADM1, UBR3, COBLL1, SLA-DQA1, IL4I1, EBP, DUOXA2, ADRB1, ABCB1, ADCY7, HIST1H2BK, MAL2, LCP2, OLFM3, OCRL, PTPRCAP, CD68, MAP4K1, LAT2, GDAP1L1, RASGRP1, CD48, AOX1, GIMAP1, MCF2L, AP1S3, SRGN, APRIL, SCARNA13, ST8SIA5, COBL, LIMA1, LIMA1, CACNA2D2, CITED1, LPPR3, HMGCS1, MME, FABP, HMGCR, FABP3, ENPP1, STRA6, FAM101A, MRO, STARD4, PAPSS2, CYB5A, AIG1, FKBP6, OLFM2, CLIC6, EMR4, SMARCA1, DUOXA1, ABCB1, RDH12, LAIR1, PANK3, NCCRP1, IGLV-7, IGKV-7, PLA2G3, IGKC, IGLV-8, IGLV-9, PCDH15, CYP17A1, ALOX15, LRP4, FMO1, ERO1B, TREM2, TCN1, SNTB1, TREM2, CLEC5A, C16orf54, FSTL4, SC4MOL, SMPDL3A, CD36, C5AR1, APOE, NCKAP1, LPXN, FN1, TLR9, IGLV-3, IBSP, CYB561, HSPB3, GCG, PTPRN, TMPRSS7, DUOX2, SV2C, LPXN, TSLP, FAM134B, SCG2, LHCGR, MAFB, VGF, LHCGR, OLFM1, TNC, CEMIP, ARHGAP30, MEST, NKG7, CYP7A1, ARL11, ZP3, SORD, SKM, STRIP2, UGT8, SLC5A2, BCKDHB, KCNE1, TRPV2, ITPR1, TIMP1, HK3, ITPR1, ITPR1, MEDAG, ITPR1, PTGFR
Sub-network (Oviduct)	69	SDF2L1, SYNDIG1, ZNF385C, PEBP4, CA12, PCDH10, COL28A1, WNT2, BRINP1, EV15, OATV1, SLC38A11, COL1A1, SCRG1, LBP, COL1A1, ABCC4, COL1A1, BRINP1, COL1A2, CRELD2, FAM3B, FAM3B, HAPLN3, KCNG1, SNCG, GPC3, FBLN2, FBLN2, UBTFL1, CPXM1, C1QL4, RCAN2, FBXO2, GPX6, GRM4, C16orf89, UPK3B, SLC2A8, SPDEF, CASQ1, CNIH2, ACTA1, CENPW, SAA2, CKM, COL3A1, MFSD2A, OLFM4, C1QTNF6, CREB3L4, EPHB1, IGF1, LMAN1L, CA3, TP53INP2, MYH7, REXO2, PEG10, TNNI1, HSD17B11, ACTC1, DERL3, C14H10orf116, AGTR2, AQP5, MYH13, SAA1, BTBD17

Sub-network (Core)	65	ACOT7, AGTR2, AOX1, AQP5, BCKDHB, CA2, CD209, CRELD2, CYP17A1, DOCK8, DPPA5, WNT5B, TMEM170B, MOCOS, PDZK1IP1, GRHPR, PEG10, PEG10, GRIA1, PAK3, ITPR1, ITPR1, NBL1, UGT8, FBLN2, ITPR1, ITPR1, FSTL4, FDPS, FKBP6, HK3, HSD17B11, ISOC1, JPH4, L1CAM, MRPS36, NANOS1, NCF1, OAT, OLFM3, PAPSS2, PCDH10, PCOLCE2, PENK, PTPRCAP, PYCARD, SBSN, SDF2L1, SGPP2, SLC27A6, SLC2A8, SLC31A2, SLC38A11, SOX7, SPDEF, STARD4, TIMP1, TLR4, TMEM86A, TNFRSF9, TSPO, VCAN, WISP2, WWP2, ZAR1
